# Are Anticholinergic Symptoms a Risk Factor for Falls in Older General Practice Patients With Polypharmacy? Study Protocol for the Development and Validation of a Prognostic Model

**DOI:** 10.3389/fphar.2020.577747

**Published:** 2021-01-14

**Authors:** Truc Sophia Dinh, Ana Isabel González-González, Andreas D. Meid, Kym I. E. Snell, Henrik Rudolf, Maria-Sophie Brueckle, Jeanet W. Blom, Ulrich Thiem, Hans-Joachim Trampisch, Petra J. M. Elders, Norbert Donner-Banzhoff, Ferdinand M. Gerlach, Sebastian Harder, Marjan van den Akker, Paul P. Glasziou, Walter E. Haefeli, Christiane Muth

**Affiliations:** ^1^ Institute of General Practice, Goethe-University Frankfurt, Frankfurt, Germany; ^2^ Red de Investigación en Servicios de Salud en Enfermedades Crónicas (REDISSEC), Madrid, Spain; ^3^ Department of Clinical Pharmacology and Pharmacoepidemiology, University Hospital Heidelberg, Heidelberg, Germany; ^4^ Centre for Prognosis Research, School of Medicine, Keele University, Staffordshire, United Kingdom; ^5^ Department of Medical Informatics, Biometry and Epidemiology, Ruhr University Bochum, Bochum, Germany; ^6^ Department of Public Health and Primary Care, Leiden University Medical Center, Leiden, Netherlands; ^7^ Chair of Geriatrics and Gerontology, University Clinic Eppendorf, Hamburg, Germany; ^8^ Department of Geriatrics, Immanuel Albertinen Diakonie, Albertinen-Haus, Hamburg, Germany; ^9^ Amsterdam UMC, General Practice and Elderly Care Medicine, Amsterdam Public Health Research Institute, Vrije Universiteit Amsterdam, Amsterdam, Netherlands; ^10^ Department of General Practice/Family Medicine, Philipps University Marburg, Marburg, Germany; ^11^ Institute of Clinical Pharmacology, Goethe-University Frankfurt am Main, Frankfurt am Main, Germany; ^12^ Faculty of Health Sciences and Medicine, Bond University, Robina, QLD, Australia; ^13^ Department of General Practice and Family Medicine, Medical Faculty OWL, University of Bielefeld, Bielefeld, Germany

**Keywords:** aged [MesH], anticholinergic burden, accidental falls [MeSH], general practice, prediction model, prognosis research, multimorbidity [MeSH], polypharmacy

## Abstract

**Background**: Cumulative anticholinergic exposure, also known as anticholinergic burden, is associated with a variety of adverse outcomes. However, studies show that anticholinergic effects tend to be underestimated by prescribers, and anticholinergics are the most frequently prescribed potentially inappropriate medication in older patients. The grading systems and drugs included in existing scales to quantify anticholinergic burden differ considerably and do not adequately account for patients’ susceptibility to medications. Furthermore, their ability to link anticholinergic burden with adverse outcomes such as falls is unclear. This study aims to develop a prognostic model that predicts falls in older general practice patients, to assess the performance of several anticholinergic burden scales, and to quantify the added predictive value of anticholinergic symptoms in this context.

**Methods**: Data from two cluster-randomized controlled trials investigating medication optimization in older general practice patients in Germany will be used. One trial (RIME, n = 1,197) will be used for the model development and the other trial (PRIMUM, n = 502) will be used to externally validate the model. A priori, candidate predictors will be selected based on a literature search, predictor availability, and clinical reasoning. Candidate predictors will include socio-demographics (e.g. age, sex), morbidity (e.g. single conditions), medication (e.g. polypharmacy, anticholinergic burden as defined by scales), and well-being (e.g. quality of life, physical function). A prognostic model including sociodemographic and lifestyle-related factors, as well as variables on morbidity, medication, health status, and well-being, will be developed, whereby the prognostic value of extending the model to include additional patient-reported symptoms will be also assessed. Logistic regression will be used for the binary outcome, which will be defined as “no falls” vs. “≥1 fall” within six months of baseline, as reported in patient interviews.

**Discussion**: As the ability of different anticholinergic burden scales to predict falls in older patients is unclear, this study may provide insights into their relative importance as well as into the overall contribution of anticholinergic symptoms and other patient characteristics. The results may support general practitioners in their clinical decision-making and in prescribing fewer medications with anticholinergic properties.

## Introduction

Medications with anticholinergic (ACh) properties are commonly used for a variety of indications (Jessen et al., 2010; Wawruch et al., 2012; Mate et al., 2015) and, along with sedatives, are the most frequently prescribed potentially inappropriate medications (PIM) in older adults (Hukins et al., 2019). Depending on patient population and setting, the prevalence of ACh drug use varies between 27% in community-based elderly patients (Ness et al., 2006) and up to 80% in more vulnerable populations such as older patients receiving home health care services or nursing home patients with dementia (Chatterjee et al., 2010; McNeely et al., 2013).

The terms “anticholinergic symptoms,” “anticholinergic side-effects,” “anticholinergic adverse effects” and “adverse drug reactions” have been used interchangeably to describe peripheral and central effects associated with anticholinergic drug use, including, for example dry mouth, dry skin, blurred vision, and drowsiness (Ness et al., 2006; Mintzer and Burns, 2016; Welsh et al., 2018). Moreover, anticholinergics have been associated with a variety of adverse outcomes, the most important of which include falls and quality of life, as well as cognitive and functional decline (Cardwell et al., 2015; Welsh et al., 2018). Falling, in particular, is a significant public health issue and represents the leading cause of disability and death in older age. It is further associated with an increase in mortality, morbidity, hospital admissions and costs for treatments (National Institute for Health and Care Excellence, 2013; Verma et al., 2016). The prevention of falls is therefore highly relevant and should be addressed by healthcare professionals involved in the care of older patients (National Institute for Health and Care Excellence, 2013). Gait and balance disorders, muscle weakness, and cognitive impairment all increase the risk of falling with advanced age (Deandrea et al., 2010). The high volume of ACh drug prescriptions raises concerns in terms of appropriateness and patient safety. This is especially true in older age, as it is associated with an increased likelihood of multiple chronic conditions (multimorbidity) and subsequent long-term medication use (polypharmacy), which may further lead to an accumulation of effects and result in substantial harm (Thomas and Brennan 2000; Hilmer et al., 2007a; Delafuente, 2008; Gnjidic et al., 2012; Kouladjian O’Donnell et al., 2017).

Salahudeen et al. have shown that older patients taking multiple ACh agents are 3.21 times more likely to experience ACh symptoms than those taking only one (Salahudeen et al., 2015b), underlining the potential risk of accumulating ACh effects, also known as ACh burden. However, ACh burden tends to be underestimated by general practitioners (GPs) (Magin et al., 2015). As a recent review of systematic reviews identified at least 18 different scales that have been developed and are used in a variety of clinical settings (Welsh et al., 2018), such underestimation may reflect the lack of an universal approach to quantifying ACh burden. These scales not only differ considerably in the way they are conducted and the (number of) included drugs, but also in their grading systems (Salahudeen et al., 2015a; Villalba-Moreno et al., 2016; Mayer et al., 2017; Welsh et al., 2018). For example, quetiapine is rated as having high or low activity, depending on the scale (Salahudeen et al., 2015a). Furthermore, the scales were developed based on expert opinions (Villalba-Moreno et al., 2016; Welsh et al., 2018) and with two exceptions (Hilmer et al., 2007b; Klamer et al., 2017), do not consider drug dose, or adequately account for patients’ individual characteristics such as sex, age, morbidity, and individual susceptibility to medications (Cardwell et al., 2015; Mayer et al., 2015).

The ability of scales to link ACh burden with numerous adverse outcomes has been inconsistently described in a variety of studies (Cardwell et al., 2015; Salahudeen et al., 2015a; Villalba-Moreno et al., 2016; Mayer et al., 2017; Welsh et al., 2018). A recent review of systematic reviews identified 62 original articles that focused mainly on the outcomes cognitive function, physical function, mortality, and delirium (Welsh et al., 2018). Little research has been undertaken to determine the predictive ability of ACh scales in relation to falls. The few studies that exist on this issue have generally involved vulnerable patient populations [e.g., from nursing homes and hospitals (Aizenberg et al., 2002; Landi et al., 2014; Vincentis et al., 2020)]. Findings from community-based settings or from general practice are not only scarce but also show inconsistent results (Marcum et al., 2015; Richardson et al., 2015; Zia et al., 2016). Furthermore, these trials, as well as ACh scales in general, were mainly conducted outside Germany (Carnahan et al., 2006; Hilmer et al., 2007b; Wilson et al., 2011; Dauphinot et al., 2014; Villalba-Moreno et al., 2016). It therefore remains unclear whether such scales are useful in clinical practice and whether they support clinical decision-making in the prescription of medications with ACh properties, for example in a German healthcare context.

The aims of this study are:

1. To develop and validate a prognostic model that considers patients’ individual characteristics in predicting the risk of falls in older general practice patients.
2. To assess the performance of several ACh burden scales in this context and to identify the one that best predict falls, and
3. To quantify whether the addition of patient-reported ACh symptoms improves the model’s predictive performance.

## Methods

### Source of Data

Data from the international PROPERmed study (PROSPERO ID: CRD42018088129) will be used to develop the model. PROPERmed includes individual participant data from five cluster-randomized controlled trials (cRCTs) conducted in German and Dutch general practices that were combined to form a harmonized database for modelling purposes. cRCTs included in PROPERmed aimed to optimize medication in older chronically ill patients from general practices. Based on the core dataset that included variables on socio-demographics, well-being, morbidity, and medication, two prognostic models were developed that predict deterioration of health-related quality of life (González-González et al., 2020) and all-cause hospitalization (Meid et al., submitted). Details on the rationale and conduct of PROPERmed will be described elsewhere (González-González et al., re-submitted). To address the research question in our study, other variables in addition to PROPERmed’s core dataset are needed from the trials, which include variables on falls as well as on ACh symptoms (e.g., vertigo/dizziness, dry mouth). To avoid systematically missing variables, only trials that are able to provide the set of additional variables will be considered for our study. Therefore, we will only consider data from the two trials RIME (Thiem et al., 2020) and PRIMUM (Muth et al., 2018) for model development. Both cRCTs were conducted in German general practices and aimed to optimize medication in older chronically ill patients from this setting. The main characteristics of PRIMUM and RIME are provided in Table 1.

**TABLE 1:** Main characteristics of the included trials.

	PRIMUM	RIME
Title	PRIoritizing MUltimedication in Multimorbidity	Reduction of potentially inadequate medication in the Elderly
Trial registration	ISRCTN99526053, NCT01171339	DRKS00003610
Study region	Hesse (Germany)	Witten/Hanover (Germany)
Start-end	2010–2012	2012–2014
Design	2-arm parallel CRT	2-arm parallel CRT
Setting	General practices (N = 72)	General practices (N = 139)
Study population	N = 502	N = 1,197
	≥60 years	≥70 years
	≥3 chronic conditions	≥6 chronic prescriptions
	≥5 chronic prescriptions	
Intervention	Structured medication review	Structured medication review
Data collection	0, 6, and 9 months	0, 6, and 12 months
No. of patients with ≥1 fall at 6-month follow-up	75	202

CRT, cluster-randomized trial.

### Participants

Participants in both trials were older patients from German general practices. In PRIMUM, 502 participants aged ≥60 years with ≥3 chronic conditions and ≥5 chronic prescriptions were included. RIME included 1,197 patients aged ≥70 years with ≥6 chronic prescriptions. To take part in the trials, participants had to provide written informed consent and be capable of participating in telephone interviews and understanding the provided information. Patients with dementia or cognitive impairment and patients with a life expectancy of less than six months (RIME) or 12 months (PRIMUM) were excluded from participation.

### Outcome

Based on the data provided in patient interviews, the study outcome of interest will be a binary indicator defined as “no falls” vs. “≥1 fall” within six months of baseline as reported in patient interviews. Patient interviews were conducted on the telephone by trained staff, whereby information concerning falls was gathered by asking the following question: “In the past six months, have you slipped, stumbled, or fallen, such that you lost your balance and landed in a lower position or on the ground?”

### Predictors

Research has identified numerous predictors for falls in the elderly such as socio-demographics (e.g. age, sex), physiological, and morbidity-related factors (e.g., gait and balance problems, dementia, depression), environmental factors (e.g., home, footwear), and medication-related factors (Deandrea et al., 2010; Ambrose et al., 2013; Zia et al., 2015; Sousa et al., 2017). Rather than include such a wide range of variables, we will follow recommendations on variable selection in prediction models that they should be clearly defined, available in medical practice, and measured in a reproducible and standardized way to improve the applicability and predictive ability of the model in new individuals (Moons et al., 2012b; Hendriksen et al., 2013; Harrell 2019). If the number of outcomes in the dataset is limited, the authors further recommend restricting the number of predictors in order to avoid overfitting the model to the data and erroneously including predictors in the model (Hendriksen et al., 2013; Moons et al., 2012b). The candidate predictors considered for our model will therefore be predefined based on a) a literature review, b) predictor availability in the included trials (RIME and PRIMUM), and c) clinical reasoning. The full list of candidate predictors and how they were measured is provided in Table 2. All predictor information was collected at baseline and includes sociodemographic and lifestyle-related factors, variables on morbidity, health status, well-being, symptoms and medication. The analyses will only consider medication with systemic effect. In accordance with Brueckle et al. (Brueckle et al., 2020), the following symptoms available in the RIME trial are considered to be anticholinergic drug reactions and will be included as potential predictors in our analyses: dizziness/vertigo, stomach pain, problems urinating, dry mouth, itching, constipation, drowsiness/fatigue. ACh burden, in particular, will be measured using five differenct scales/equations with the aim of comparing their ability to predict falls. An overview of ACh scales’ characteristics is presented in Table 3. For our study, we have selected the scales that were found to be frequently used in the review of systematic reviews examining the association between ACh burden and patient-relevant outcomes published by Welsh et al. (2018): the Anticholinergic Drug Scale (ADS) (Carnahan et al., 2006), the Anticholinergic Risk Scale (ARS) (Rudolph et al., 2008) and the Drug-Burden-Index (DBI) (Hilmer et al., 2007b). We will further include the Muscarinic Acetylcholinergic Receptor ANTagonist Exposure Scale (MARANTE) (Klamer et al., 2017), as this scale combines potency with the dosage spectrum as well as the German Anticholinergic Burden Score (Ger-ABS) (Kiesel et al., 2018), as it is the only scale that was explicitly developed for the German drug market. In the two studies RIME and PRIMUM, prescribed drugs were coded based on the Anatomic Therapeutic Chemical (ATC) classification. For our analyses, ACh burden will be calculated in accordance with the scales’ definitions, and respective ATC codes for the German drug market will be identified accordingly. For the calculation of the DBI, the minimum recommended daily dose as listed by the US Food and Drug Administration (FDA) is required (Kouladjian et al., 2014) As minimum recommended daily doses vary between countries, we will use a version of the DBI that was adapted for the German drug market in the COFRAIL-study by Thürmann et al. (German Innovation Funds No. 01VSF17053). Candidate predictors collected as continuous measurements will be kept continuous in the analyses. We will assume continuous candidate predictors to be linearly associated with the outcome with the exception of ACh variables, for which research has shown that high ACh load appears to reach a plateau, indicating a non-linear relationship (Kersten et al., 2013; Mayer et al., 2015).

**TABLE 2:** Candidate predictors available in the development dataset.

Group	Type of predictor	Candidate predictor	Data collection	Data type	Measurement unit
A	Sociodemographic and lifestyle-related	Intervention status	Registration form	Cat	Intervention, control
		Age	Registration form	Cont	Years
		Sex	Registration form	Cat	Male, female
		Living situation	Patient interview	Cat	Home, institutionalized
		Educational	CRF	Cat	Low, medium, high
		Smoking	Patient interview	Cat	Smoker, ex-smoker, non-smoker
B	Morbidity-related	Hypertension	Patient interview	Bin	No, yes
		Diabetes mellitus	Patient interview	Bin	No, yes
		Coronary heart disease	Patient interview	Bin	No, yes
		Osteoarthritis	Patient interview	Bin	No, yes
		COPD/asthma	Patient interview	Bin	No, yes
		Vision problems	Patient interview	Bin	No, yes
		Hearing problems	Patient interview	Bin	No, yes
		Cancer	Patient interview	Bin	No, yes
		Heart failure	Patient interview	Bin	No, yes
		Cerebrovascular disease	Patient interview	Bin	No, yes
		Osteoporosis	Patient interview	Bin	No, yes
		Depression	Patient interview	Bin	No, yes
		Rheumatoid/seropositive arthritis	Patient interview	Bin	No, yes
		Atherosclerosis/peripheral vascular disease	Patient interview	Bin	No, yes
		Parkinsonism	Patient interview	Bin	No, yes
		HIV/AIDS	Patient interview	Bin	No, yes
		Lipid disorder	Patient interview	Bin	No, yes
		Gout	Patient interview	Bin	No, yes
		Thyroid disorders	Patient interview	Bin	No, yes
		Gastric or duodenal ulcer	Patient interview	Bin	No, yes
		Liver disorder	Patient interview	Bin	No, yes
		Urinary disease	Patient interview	Bin	No, yes
		Anemia	Patient interview	Bin	No, yes
		No. of chronic conditions	Patient interview	Cont	No, yes
C	Health status and well-being related	Pain	Patient interview	Bin	No, yes
		Quality of life (EuroQol Group 1990)	Patient interview	Cont	Score
		Functional status (Saliba et al., 2001)	Patient interview	Cont	Score
		Cognitive function	Patient interview	Cont	Score
		All-cause hospital admissions	Patient interview	Bin	No, yes
		History of falls	Patient interview	Cat	≤1 fall, ≥ 2 falls
D	Medication related	No. of drugs	Patient interview	Cont	Frequency
		ACh burden (Carnahan et al., 2006; Hilmer et al., 2007b; Rudolph et al., 2008; Klamer et al., 2017; Kiesel et al., 2018)	Calculated using medication data from patient interview	Cont	Weighted index
E	Symptoms	No. of symptoms	Patient interview	Cont	Frequency
		Dizziness/vertigo	Patient interview	Bin	No, yes
		Problems urinating	Patient interview	Bin	No, yes
		Stomach pain	Patient interview	Bin	No, yes
			Patient interview	Bin	No, yes
		Drowsiness/fatigue^a^	Patient interview	Bin	No, yes
		Dry mouth^a^	Patient interview	Bin	No, yes
		Itching^a^	Patient interview	Bin	No, yes
		Constipation^a^	Patient interview	Bin	No, yes

ACh, anticholinergic; Bin., binary; Cat., categorial; Cont., continuous; COPD, chronic obstructive pulmonary disease; CRF, case report form; HIV/AIDS, human immunodeficiency virus/acquired immune deficiency syndrome; No, number.

^a^ Symptoms only available in the development dataset (RIME).

**TABLE 3:** Overview of anticholinergic scales’ characteristics.

Study (year)	Scale	Country	No of included drugs	Grading system	Dosage considered
Carnahan et al. (2006)	Anticholinergic drug scale (ADS)	USA	117	Scores: 0–3	No
Klamer et al. (2017)	Muscarinic acetylcholinergic receptor ANTagonist exposure scale (MARANTE)	Belgium	41	Equation	Yes
Hilmer et al. (2007b)	Drug-burden-index (DBI)	Australia	128	Equation	Yes
Rudolph et al. (2008)	Anticholinergic risk scale (ARS)	USA	49	Scores: 0–3	No
Kiesel et al. (2018)	German anticholinergic burden score (Ger-ABS)	Germany	151	Scores: 0–3	No

### Sample Size

When designing a prediction model study, Riley et al. (2019) recommend that the minimum sample size is calculated to meet certain criteria and minimize overfitting. As we will retrospectively analyze individual participant data, the sample size and prevalence are already given by the included trials. The *pmsampsize* package in R provided by the authors will be used to calculate whether our sample size meets the suggested criteria, and, in view of the number of predictors, to determine the level of expected overfitting of our model (Ensor et al., 2019). We will use data from the RIME study for model development because of its larger sample size (n = 1,197 vs. n = 502) and larger number of events (n = 202 vs. n = 75) compared to PRIMUM.

### Missing Data

If a predictor is not present in either of the included studies, it will be considered systematically missing for that study. For practical reasons, systematically missing variables will not be imputed and will therefore not be considered candidate predictors in model development.

Because the exclusion of participants with missing values from analyses (as in complete case analyses) reduces the effective sample size and may lead to biased estimates, we will follow recommendations on dealing with the missing values that inevitably occur in predictors and use multiple imputation (MI) techniques to impute partially missing data (Moons et al., 2012b; Harrell 2015). In the first stage of MI, multiple datasets will be created and the missing values imputed based on the observed data and on the assumption that data are missing at random. The imputation will then be repeated multiple times to properly account for variability in the imputation process. As an approximation, the number of imputed datasets (*m*) will correspond to the percentage of incomplete observations (White et al., 2011). The analyses will be performed for each dataset, resulting in *m* analysis results. The last step of MI is a pooling step, whereby overall estimates will be obtained using Rubin’s rules (White et al., 2011; Harrell 2015). In line with the reporting suggestions of Sterne et al., we will compare results from complete cases compared with results based on MI in order to be able to detect and understand differences between them (Sterne et al., 2009).

### Statistical Analysis Methods

All statistical analyses will be conducted using R version 4.0 (R Foundation for Statistical Computing, Vienna, Austria).

#### Model Development, Performance and Internal Validation

**FIGURE 1:**
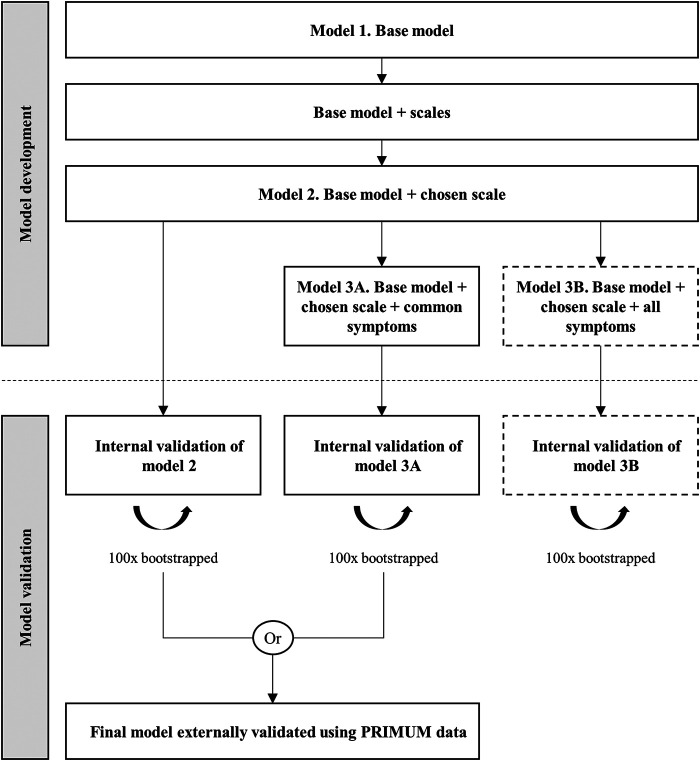
Model development and validation process.

The model will be built as follows (see Figure 1).

##### Model 1. Base Model

First, multivariable logistic regression will be used to develop a base model that employs data from the RIME trial to predict falls within six months of baseline. To find the best combination of candidate predictors (groups A to C), the multivariable fractional polynomial (MFP) approach will be used. The MFP approach is a procedure that builds the model, while simultaneously determining a suitable functional form for continuous variables. It starts with a full model that includes all candidate predictors and removes the least significant variables via backward elimination. The choice of predictors for elimination will be based on Akaike’s Information Criterion (AIC) (Sauerbrei et al., 2020).

##### Model 2. Base Model + Scale

Secondly, we will add variables on ACh burden (group D) to the base model. Here, we aim to compare different models for each of the four scales and one equation that measure ACh burden (Carnahan et al., 2006; Hilmer et al., 2007b; Rudolph et al., 2008; Klamer et al., 2017; Kiesel et al., 2018). We will assess the apparent performance of the models for discrimination using the *c*-statistic [equal to the area under the receiver operating characteristic curve (ROC)] and the model fit using AIC. The preferred model will be the one with the lowest AIC and the highest *c*-statistic. A *c*-statistic close to one indicates excellent discrimination and 0.5 indicates that the model cannot discriminate between individuals that have the outcome and those that do not (Steyerberg and Vergouwe 2014).

##### Model 3. Base Model + Chosen Scale + Symptoms

In a third step, we will extend the model by adding symptom variables (group E) and quantify whether adding these variables to the developed model is beneficial. The development and validation datasets have three symptoms in common: dizziness/vertigo, problems urinating, and stomach pain. The development dataset includes four further symptoms: drowsiness/fatigue, dry mouth, itching, and constipation (see Table 2). We will therefore develop two different models at this stage: model *3A* (Base model + chosen scale + *common* symptoms), which will be considered for external validation, and model *3B* (Base model + chosen scale + *all* symptoms), which will be developed to conduct an exploratory assessment of whether the additional variables considerably improve predictive performance.

Prior to comparing performance, models *2*, *3A*, and *3B* will be internally validated using bootstrapping. This approach includes: 1) generating a bootstrap sample, 2) developing the model using that bootstrap sample, 3) determining the predictive performance of the model in the bootstrap and the original samples, 4) calculating optimism as the difference in performance between bootstrap and the original samples for each performance measure, 5) repeating steps 2 to 4, 100 times, and 6) averaging the estimates of optimism and subtracting average optimism from the apparent performance of the original model in the original dataset to obtain optimism-adjusted performance estimates (Moons et al., 2015). If necessary, we will shrink coefficients to correct for overfitting.

After internal validation, we will compare the performance and discriminative ability of the models before and after adding the variables on anticholinergic symptoms (model *2* vs. model *3A*). In case of high collinearity between the predictors, we will assume that adding symptoms will not improve model performance (Harrell 2015). We will compare the *c*-statistics of the models and determine the change in AIC (Moons et al., 2012a; Cook 2018). The model with the higher *c*-statistic and lower AIC will be selected for external validation. Because the validation dataset does not include all symptoms contained in model *3B* (*base model* + *chosen scale* + *all symptoms*), this model will not be considered for external validation. However, the use of internal validation to compare the performance of models *3A* and *3B* may indicate whether the additional symptoms considerably improve predictive performance.

#### External Validation

Using the PRIMUM dataset, we will externally validate the final model and assess discriminative ability by estimating the observed/expected ratio (comparing the predicted number of falls from our final model with the observed number of falls in PRIMUM) and the *c*-statistic. We will further calculate the calibration slope, calibration-in-the-large, and produce a calibration plot (Steyerberg et al., 2010).

## Anticipated Results

Our results are expected to contribute to the ongoing discussion regarding the use of ACh scales in clinical practice. Analyses may identify differences in the scales’ ability of linking ACh burden with falls for older general practice patients, indicating their usefulness in clinical practice in a German health care context. Patients’ individual characteristics and patient-reported symptoms may reflect clinically relevant and feasible indicators to identify patients from a heterogeneous population that might be at high risk of experiencing falls.

### Strengths

As one of the limitations of ACh scales’ is their failure to take into account individual patient characteristics, patient-reported symptoms may function as a surrogate for patient susceptibility to such medications and may therefore help clinicians identify patients at high risk of falls. Furthermore, a prognostic model tends to perform optimistically when using the data from which it was derived. It is therefore necessary to assess its accuracy by externally validating it using a separate dataset (Moons et al., 2015). External validation will enable to demonstrate its predictive value and generalizability in a similar population but with different individuals (Steyerberg and Vergouwe, 2014).

### Limitations

One limitation is the risk of recall-bias in the patients’ ability to self-report falls (Gillespie et al., 2012). A verification of patient-reported falls in electronic medical records (EMR) was not possible in the studies because are not systematically documented in EMRs. The eligibility criteria and availability of data will not allow us to include some of the known risk factors for falls in the elderly such as dementia, muscle strength, or gait problems (Deandrea et al., 2010; Ambrose et al., 2013; Sousa et al., 2017). It will also prevents us from considering the known increased risk of inter-individual variability in older age that results from, for example, age-related pharmacokinetic and pharmacodynamic changes and genetic variation in metabolism (Nishtala et al., 2016). Furthermore, we are not able to prove the causality of the symptoms, as they may have been caused by factors other than a (anticholinergic drug) reaction to the medication. Nevertheless, as patients’ susceptibility varies considerably, we believe that taking patient-reported symptoms into consideration will provide additional decision support to the mere calculation of anticholinergic load (Kersten and Wyller, 2014). As external validation will be carried out retrospectively using an existing dataset, the 75 events from PRIMUM will not meet the suggested minimum of 100 events required to externally validate a prognostic model (Collins et al., 2016). In consequence, we expect estimates of performance measures to have relatively wide 95% confidence intervals. In view of this limitation, we will carefully discuss and interpret our findings.

## Discussion

Literature on ACh scales’ ability to predict falls is scarce. This study may therefore provide insights into the issue by comparing the measurements provided by several scales with the number of falls and determining whether the inclusion of patient-reported symptoms is beneficial in this context. Our results may therefore support GPs with their clinical decision-making in reducing medications with ACh properties in older patients.

## Data Availability Statement

The raw data supporting the conclusions of this article will be made available by the authors, without undue reservation. Requests to access these datasets should be directed to dinh@allgemeinmedizin.uni-frankfurt.de.

## Ethics Statement

The studies involving human participants were reviewed and approved. The Ethics Commission of the Medical Faculty of the Johann Wolfgang Goethe University, Frankfurt/Main approved the PRIMUM study (date: 20/05/2010, reference: E46/10). The Ethics Commission of the University Witten/Herdecke approved the RIME study (date: 28.02.2012, reference: 147/2011). The Ethics Commission of the Medical Faculty of the Goethe University, Frankfurt/Main confirmed that no extra vote was necessary for the anonymous use of data within the PROPERmed study (13/07/2017) because included trials were approved by their ethics commissions separately. The patients/participants provided their written informed consent to participate in this study.

## Author Contributions

TD, PG and CM initiated and designed the study. TD drafted the manuscript. AG-G, AM, JB, H-JT, HR, KS, M-SB, MA, ND-B, PE, SH, UT, and WH provided methodological guidance. All authors contributed to the revision of the manuscript. All authors read and approved the final manuscript.

## Funding

PROPERmed was funded by the German Innovation Fund (grant number 01VSF16018). RIME was funded by the German Federal Ministry of Education and Research (grant number 01ET1005A). PRIMUM was funded by the German Federal Ministry of Education and Research (grant number 01GK0702). The funders had no role in study design, data collection and analysis, decision to publish, or preparation of the manuscript.

## Conflict of Interest

TD, AG-G, AM, HR, JB, H-JT, PE, FG, MA, WH, and CM report grants from the German Innovation Fund during the conduct of the PROPERmed study (grant number 01VSF16018). KS reports grants from NIHR School for Primary Care Research during the conduct of the study. KS is funded by the National Institute for Health Research School for Primary Care (NIHR SPCR Launching Fellowship). The views expressed are those of the author(s) and not necessarily those of the NIHR or the Department of Health and Social Care. UT reports grants from the German Federal Ministry of Research and Eductation during the conduct of the RIME study (grant number 01ET1005A). UT has received travel expenses from the German Society of Internal Medicine and the Goethe-University Frankfurt/Main for scientific meetings. UT has received travel expenses and an honorarium for a lecture from the Foundation for Quality and Efficiency in Health Care (IQWIG, Germany) and for a symposium from the BANSS Foundation (Biedenkopf, Germany).

The remaining authors declare that the research was conducted in the absence of any commercial or financial relationships that could be construed as a potential conflict of interest.

## Abbreviations

ACh, anticholinergic; ADS, anticholinergic drug scale; AIC, akaike’s information criterion; ARS, anticholiergic risk scale; ATC, anatomic therapeutic chemical; cRCT, cluster-randomized controlled trials; DBI, drug burden index; EMR, electronic medical record; FDA, food and drug administration; Ger-ABS, german anticholinergic burden score; GP, general practitioner; MI, multiple imputation; MFP, multivariable fractional polynomial; PIM, potentially inappropriate medication; PRIMUM, prioritizing multimedication in multimorbidity; RIME, reduction of potentially inadequate medication in the elderly; ROC, receiver operating characteristic
